# Age dependent normative data of vertical and horizontal reflexive saccades

**DOI:** 10.1371/journal.pone.0204008

**Published:** 2018-09-18

**Authors:** Susanne Hopf, Matthias Liesenfeld, Irene Schmidtmann, Shahrzad Ashayer, Susanne Pitz

**Affiliations:** 1 Department of Ophthalmology, University Medical Center Mainz, Mainz, Germany; 2 Department of Urology, Klinikum Worms, Worms, Germany; 3 Institute of Medical Biostatistics, Epidemiology and Informatics (IMBEI), University Medical Center Mainz, Mainz, Germany; 4 Group practice Dr. med. Kirsch & Partner, Hamburg, Germany; 5 Orbital Center, Ophthalmic Clinic, Bürgerhospital Frankfurt, Germany; Faculty of Medicine, Cairo University, EGYPT

## Abstract

**Purpose:**

There is some controversy whether or not saccades change with age. This cross-sectional study aims to clarify the characteristics of reflexive saccades at various ages to establish a normative cohort in a standardized set-up. Second objective is to investigate the feasibility of saccadometry in daily ophthalmological practice.

**Methods:**

One hundred healthy participants aged between 6 and 76 years underwent an ophthalmologic examination and saccadometry, using an infrared video-oculography device, sampling at 220 Hz. The reflexive saccades were evoked in four directions and three target displacements each (5°/15°/30° horizontally and of 5°/10°/20° vertically). Saccadic peak velocity, gain (amplitude/target displacement) and latency were measured.

**Results:**

Mean peak velocity of saccades was 213°/s (± 29°/s), 352°/s (± 50°/s) and 455°/s (± 67°/s) to a target position 5°, 15°and 30° horizontally, respectively, and 208°/s (± 36°/s), 303°/s (± 50°/s) and 391°/s (± 71°/s) to a target position 5°, 10° and 20° vertically. The association between peak velocity and eccentricity proved to be present at any age in all four directions. We found no relevant effect of age on peak velocity, gain and latency in a fitted linear mixed model. However, latency becomes shorter during childhood and adolescence, while in adulthood it is relatively stable with a slight trend to increase in the elderly. Saccades are more precise when the target displacement is small. Isometric saccades are most common, followed by hypometric ones. All children and elderly were able to perform good quality saccadometry in a recording time of approximately 10 minutes.

**Conclusion:**

The presented data may serve as normative control for further studies using such a video-oculography device for saccadometry. The means of peak velocity and the gain can be used independently from age respecting the target displacement. Latency is susceptible to age.

## Introduction

Saccades are rapid conjugated eye movements -actually the fastest movements humans can perform-, and are performed to focus peripheral targets rapidly on the fovea [1]. As the fovea captures only one degree of the visual field, these movements have to be extremely precise. The high velocity is important to overcome blurry vision [2].

The main parameters that characterize reflexive saccades are latency (time interval between visual target and initiation of saccade, also called saccadic reaction time or delay), peak velocity (maximal angular distance per time during the saccade) and amplitude (angular distance). Physiologic saccades are specified with a latency of around 200 ms with considerable individual differences and a peak velocity of 400 to 800°/s [3]. The secondary parameter named gain, is a dimension for precision and is defined as the quotient of amplitude [°] and target displacement [°] [4,5]. Precision depends exclusively on pre-saccadic signal processing; visual feedback mechanisms are not fast enough to play a role [6]. The most accurate saccades, defined by a gain of 1, are rarely seen [6]. So-called isometric saccades are characterized by a gain of 0.9 to 1.1; hypometric saccades are defined as gain below 0.9, hypermetric saccades as gain over 1.1 [7]. The amplitude has an impact on the peak velocity. This correlation was named “main sequence” by Bahill et al. in 1975 [8].

Visually guided reflexive saccades were analyzed in this study. These are triggered reflexively by an unexpected target, in response to the appearance of a new visual target in the paracentral or peripheral visual field [1,6]. These reflexive saccades, also called pro-saccades, allow to evaluate basic findings such as attention and sensorimotor control [1,9,10]. By evaluating different types of saccades clinically as well as quantitatively, conclusions concerning the integrity or pathology of neuronal structures can be drawn. This is possible as the dynamic characteristics and the neurobiological origins are well understood [4,6]. As neurologic pathologies, such as Gaucher disease type 3, progressive supranuclear palsy or Niemann-Pick disease type C, can affect horizontal saccades as well as vertical saccades, we investigated both directions. However, neurodegenerative processes are supposed to be part of the ageing process of human beings. Thus, an age related change of saccadic parameters seems plausible.

There is some controversy whether or not the diverse characteristics of saccades change with age. While a number of studies could not demonstrate an age dependence of peak velocity, gain or amplitude [11–13], Irving et al. report an inverse u-shaped relation between age and peak velocity with a maximum at 14 years and an increase of hypometric saccades with increasing age and amplitude (for saccades over 20°) [14].

An age depending increase of latency (slower saccadic reaction time), however, has consistently been reported in the literature [15–19] with slower saccadic reaction times in people aged 60 to 79 whereas the fastest reaction time (low latency) was detected in people aged 20 to 30 years. In comparison, children between 5 and 8 years of age presented slow saccadic reaction times and a high intra-subject variance. This asymmetric bimodal distribution is thought to reflect physiologic childhood development and decline with older age [12]. Higher-level cognitive processes, such as visuo-spatial attention may also play a role [20,21].

It is well known that the outcome of saccadometry depends on the examination environment [22]. Therefore it is particularly important that examination conditions are well standardized. Furthermore, each laboratory should have an own data base with reference values derived from a local healthy cohort and under standardized conditions [6].

In this study, patients were measured with the EyeSeeCam (EyeSeeTec, Fürstenfeldbruck, Germany), an infrared video-oculography device with a reasonable sampling rate of 220 Hz and a short calibration time of less than two minutes. The EyeSeeCam is a relatively new device in measuring saccades horizontally and vertically. To the best of our knowledge a normative cohort with a broad and balanced age distribution up to now has not been investigated. The aim of this study is to establish a representative database of a healthy cohort and to evaluate the feasibility of the device in daily ophthalmological practice.

## Material and methods

### Subjects

One hundred and two healthy study participants were recruited. Exclusion criteria were neurological or ocular disorders with visual impairment reported by the study participants or detected during the examination. Children younger than 6 years were not included. Written consent was obtained from the participants or their legal guardians. This study was approved by the Medical Ethical Committee of the State Chamber of Medicine of Rhineland Palatinate in Mainz, Germany (reference number 837.125.15).

With 102 participants, a 95% confidence interval with a 90% coverage probability that has a length of 0.43 standard deviation was reached. A 95% confidence interval contains the population mean with probability 95%. 90% coverage probability means that 90% of the time a 95%-confidence interval has at most the specified width (0.43 SD). The study group was stratified into seven age dependent subgroups to provide an age distribution equivalent to targeted patient groups for further investigations (see Table 1). Each subgroup comprised a minimum of ten individuals.

**Table 1:** Stratification of the cohort into age-dependent subgroups.

*Age (years)*	*Number of participants*
6 to 12	13
13 to 19	12
20 to 29	29
30 tp 39	11
40 to 49	11
50 to 59	12
above 60	12
total	100

### Examination procedure

All study participants underwent a clinical and saccadometric eye examination according to a standardized investigation form. The clinical examination included: refraction, best corrected visual acuity, orthoptic assessment (stereoscopic vision, cover test, motility, optokinetic nystagmus (OKN) by drum and clinical saccade testing), anterior and posterior segment examination, optical coherence tomography of the optic nerve head and the macula. The technical test of saccadic eye movements using video-oculography included a protocol for saccades in vertical and horizontal direction and additionally an OKN protocol. Clinical examinations of all subjects were performed by the same investigator, while the technical examinations were conducted by two experienced investigators.

### Data acquisition using video-oculography

The saccades were recorded by an infrared video-oculography (VOG) device (EyeSeeCam vHIT, EyeSeeTec Fürstenfeldbruck, Germany), that was linked to a MacBook Pro 13” (OS X Version 10.9.5, Apple Inc.) equipped with the EyeSeeCam software (EyeSeeCam VOG HIT System Reversion r3429 and r3444, EyeSeeTec Fürstenfeldbruck, Germany). The EyeSeeCam provides an infrared-illumination and six depth-of field inertial sensors, a laser calibration module and a head mask resembling swimming goggles. The camera is mounted on the head mask above the left eye and records the eye movements through a transparent mirror surface. The fixation of the camera by a ball joint allows to center the pupil. The manual focus can be adjusted by a rotatable lens. The video image is sampled at 220 Hz (every 3.6 ms). The target displacements were predetermined by the software at 5°/15°/30° horizontally and 5°/10°/20° vertically.

### Examination

The study participants were seated on a height-adjustable chair, facing the center of a computer monitor (Dell Technologies Inc., 19” screen BQR-1908FPb, 1280 x 1024 resolution, 60 Hz refresh rate, 300 CD/m^2^ display luminance and 5ms image building time) at a distance of 60 cm from the glabella to the monitor. The head was stabilized by a height-adjustable chin rest, mounted on the examination table. In a dimly lit environment, the visual target on the dark monitor was a white spot or a smiley, depending on the sequence. The white spot was predetermined for calibration and short sequence studies (42 sec.) and smileys were used in the long sequence studies to maintain attention (4 min.). The short sequence may be particularly useful in cases showing moderate compliance.

### Calibration

The calibration was realized automatically by tracking the eye position at a central fixation light point and light points displayed at 10° gaze excursion (smooth pursuit) on the monitor. Only movements of the left eye were measured.

In the long sequence testing, the fixation target switched between the center and targets located ±5°, ±10° vertically and ±5°, ±15° horizontally whereby only the displacement from the central fixation point to the target was counted, not the backwards saccade. In this way, prediction or anticipation of saccades could be minimized. Also excursions of 20° vertically and 30° horizontally were measured. Each measured large saccade was counted. A total of 20 or 21 pseudorandomly distributed visual targets were displayed per direction (upwards, downwards, rightwards and leftwards) in pseudorandomly time intervals of 2.5 s. to 3 s. in the long sequence testing. The sequence is shown in the supplemental (S1 Fig).

The velocity threshold of saccade detection was set as 100°/s. Further criteria were the occurrence 0.5 s after the appearance of the target at latest and an amplitude of more than 0.5 of the relevant target displacement. The number of registered saccades was displayed and the parameters peak velocity, amplitude and latency of the counted saccades were calculated on the basis of the calibration of each participant.

### Data quality check of the video-oculography

The performance was monitored on the MacBook screen via video transmission and the raw data line (oscilloscope display) of the eye tracking. Thereby, artefacts from blinking or head movements could be seen by the investigator. Artefacts can lead to false or missing saccades and were minimized by precise instructions such as “blinking should be suppressed during saccades” or “blinking should be reserved for pauses” or “the head must be held in the same position”. In this trial, we did not have to remove saccades with blinks from the raw data line, because blinks only led to missing and not to false saccades, because we used the standard calculation and not the “special saccades calculation” analysis. Additionally, make-up was removed from the eyelashes to avoid artefacts.

A data quality check after the examination was conducted by checking the result diagrams, the measured values (peak velocity, amplitude and latency) and the number of detected saccades (N) for plausibility.

### Statistics

The software calculated the means of peak velocity, amplitude and latency for each participant, target displacement and direction. Gain was calculated post hoc. Averaging was not made for opposite directions because some pathologies can be seen in one direction only. Statistical Package for Social Sciences (SPSS 23) and Statistical Analysis System (SAS 9.4) were used for the analysis after manual data transfer. A descriptive analysis was performed for the measured values (mean, median, standard deviation, bar charts, box plots and scatter plots). The association of measured values for peak velocity, amplitude and latency with age (divided into subgroups) were displayed in box plots. To describe the dependence of the peak velocity, latency and gain respectively on age, eccentricity and direction we fitted linear mixed models with velocity, latency and gain as dependent variables, age as quantitative fixed effect, eccentricity and direction as categorical fixed effects and subject as random effect thereby taking into account that multiple observations on a subject may not be independent.

## Results

Of the 102 participants, two were excluded from statistical analysis due to poor compliance (excessive fatigue) and poor vision (logMAR visual acuity > 1.3), resulting in a final sample size of 100.

54 female and 46 male participants aged 6 to 75 years (median 28.4 years) were analyzed. Division into the seven subgroups was made as shown in Table 1.

The feasibility of saccadometry in daily ophthalmologic practice could be verified: Participants of all age groups, including children and elderly were able to perform saccadometry within reasonable time of 10 to 15 minutes, without requiring re-examination due to poor data quality, which in general was remarkably high. We hardly measured any artefacts allowing to record nearly all saccades. Deleting or redefining of saccades was not necessary in this healthy cohort. Long sequence data were taken for the analysis with merely one exception, in which we relied on the short sequence data due to moderate attention of one child. One participant in the subgroup above 60 years was under anticonvulsant medication (lamotrigine and levetirazetam) but was included for three reasons: he was the oldest participant, he had the fastest eye movements of the total cohort and his medication is a common medication in patients with Gaucher disease type 3 or Niemann-Pick disease type C. As these are diseases showing changes in saccadic eye movements, the benefit was therefore given.

Refraction revealed

- 48.5% emmetropic eyes (-0.5 dpt. to +0.5 dpt.),
- 31.5% myopic eyes (< -0.5 dpt.), 1% highly myopic eyes (≤ -6 dpt., max. -7.6 dpt.) and
- 19% hyperopic eyes (> +0.5 dpt., max. +5.5 dpt.).

Mean and median of the best corrected logMAR visual acuity was 0.0 in each eye (range 0.8 to -0.2 OD and 0.7 to -0.2 OS). Visual acuity without correction was averaged 0.13 (median 0.0) in each eye in 5 m distance and adequate to see and fixate the icons on the screen at 60 cm distance.

The prevalence of strabismus was 3%, stereoscopic vision was limited in those cases. Motility was normal in all participants as well as clinical saccade testing. Optokinetic nystagmus (OKN) was abnormally triggered vertically upwards and downwards in one participant, who otherwise had normal eye findings and normal saccadometry.

The following findings were regarded irrelevant for saccadometry: Pseudoexfoliation syndrome, pterygium, signs of laser treatment and nummuli of the cornea were found in one participant each and irrelevant myopic fundus changes in two additional participants.

Optical coherence tomography of the optic nerve head and the macula were normal.

### Basic saccade parameters: Peak velocity, gain and latency

A minimum of 4 recorded saccades (as response to 6–8 stimuli per direction and target displacement) in each of the 100 included participants was detected in 98.5% of the 12 x 100 categories. Missing values were few (8/1,200; 0.7%). The saccade parameters had an approximately normal distribution (mean values, standard deviations and ranges are listed in the supplemental, S1 Table). For better visualization, peak velocity and gain are displayed in the following box plots according to the corresponding direction of gaze and the angular distance of the target displacement.

**Fig 1:**
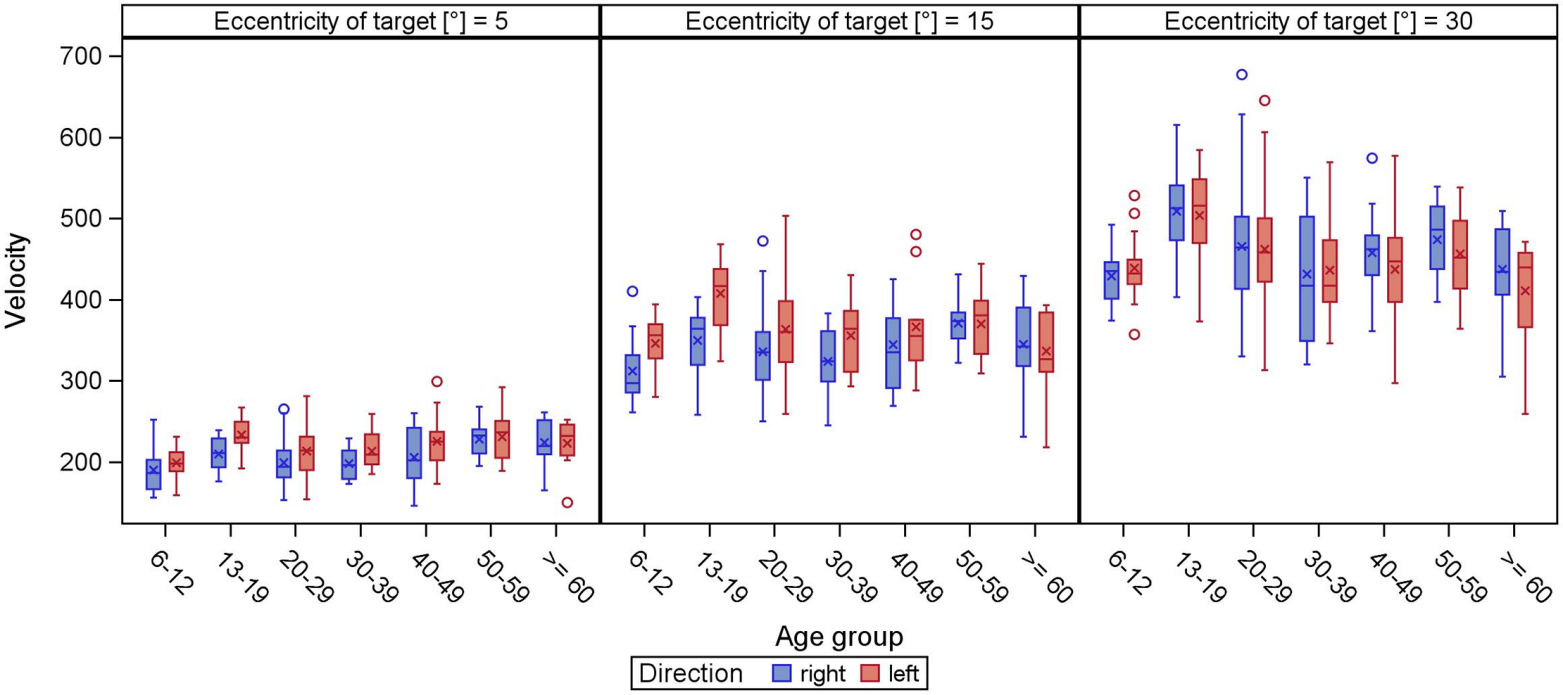
**Peak velocity of horizontal (direction right/left) reflexive saccades.** The peak velocity (y-axis) is displayed in boxplots in dependence of the age groups (x-axis) for different target eccentricities. The whiskers extend to the minimum and maximum, when there are no outliers (displayed as circles, meaning values defined by a distance of more than 1.5 times the interquartile distance from the box). The lower boundary of the box marks the 25^th^ percentile (25% quartile), the line within the box indicates the 50^th^ percentile (median) and the upper boundary represents the 75^th^ percentile (75% quartile). The arithmetic mean is shown as asterisk. In all age groups, the peak velocity increases with increasing target eccentricity.

**Fig 2:**
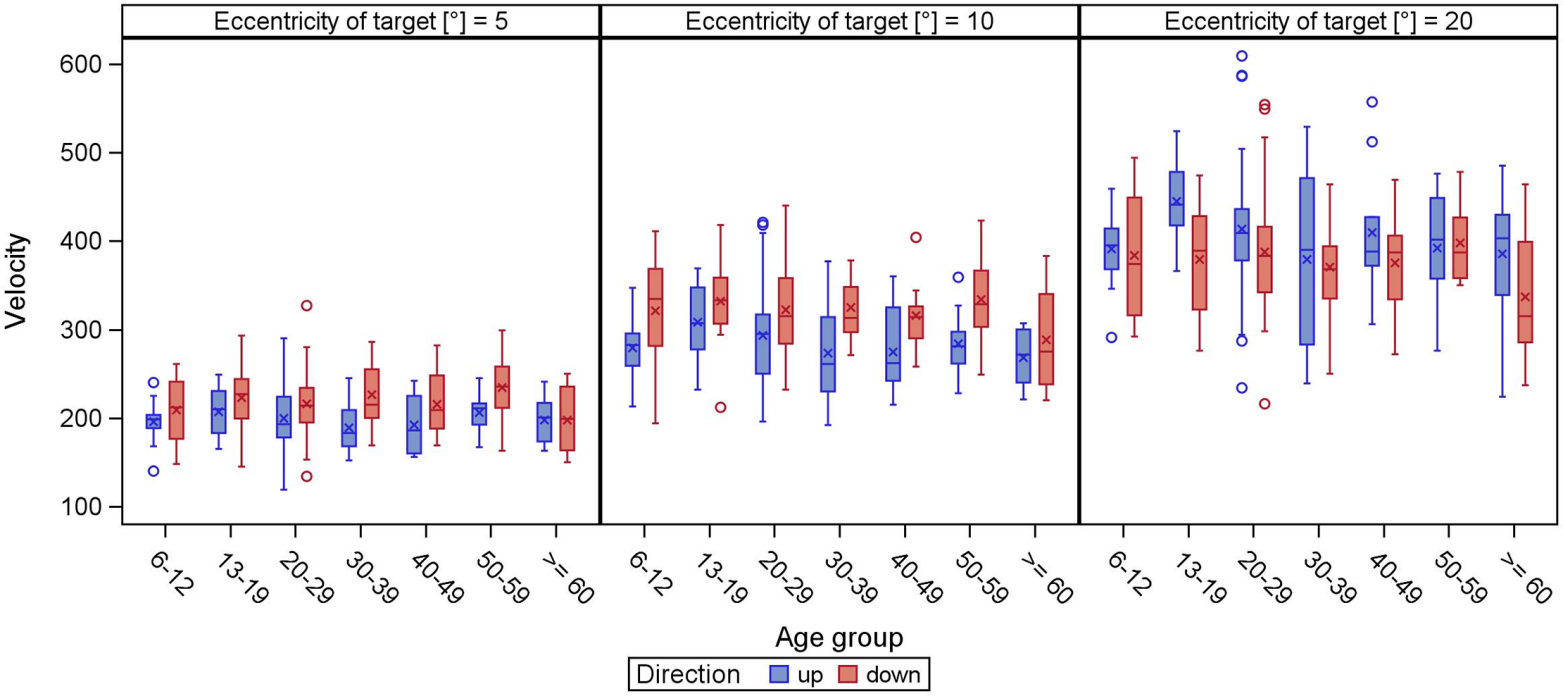
**Peak velocity of vertical (direction up/down) reflexive saccades.** The peak velocity (y-axis) is displayed in boxplots in dependence of the age groups (x-axis) for different target eccentricities. The whiskers extend to the minimum and maximum, when there are no outliers (displayed as circles, meaning values defined by a distance of more than 1.5 times the interquartile distance from the box). The lower boundary of the box marks the 25^th^ percentile (25% quartile), the line within the box indicates the 50^th^ percentile (median) and the upper boundary represents the 75^th^ percentile (75% quartile). The arithmetic mean is shown as asterisk. In all age groups, the peak velocity increases with increasing target eccentricity.

The peak velocity increased with increasing target eccentricity in any tested direction and over all age groups (Figs 1 and 2). Peak velocity of smaller target displacements was relatively stable regardless of age, while larger target displacements had the largest variability.

**Fig 3:**
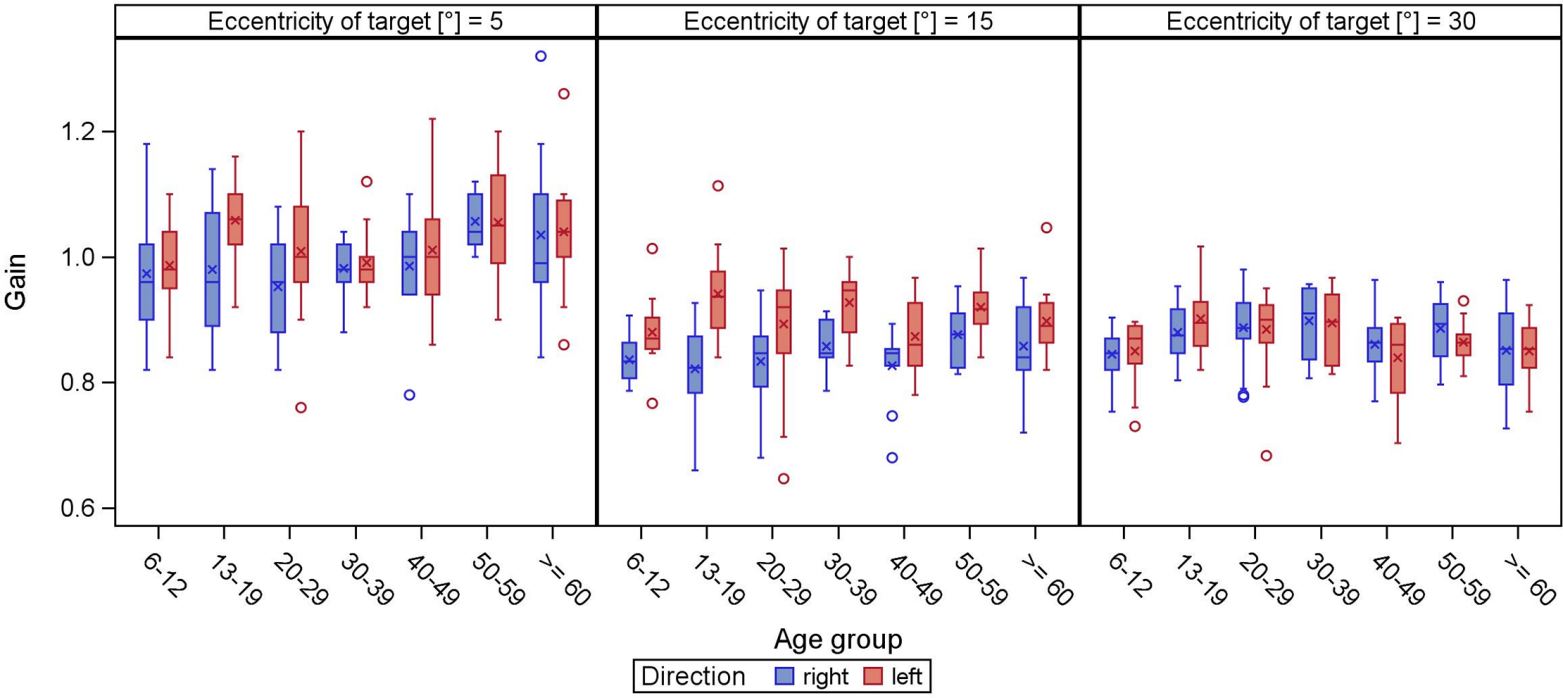
**Gain of horizontal (direction right/left) reflexive saccades.** The gain (y-axis) is displayed in boxplots in dependence of the age groups (x-axis) for different target eccentricities.

**Fig 4:**
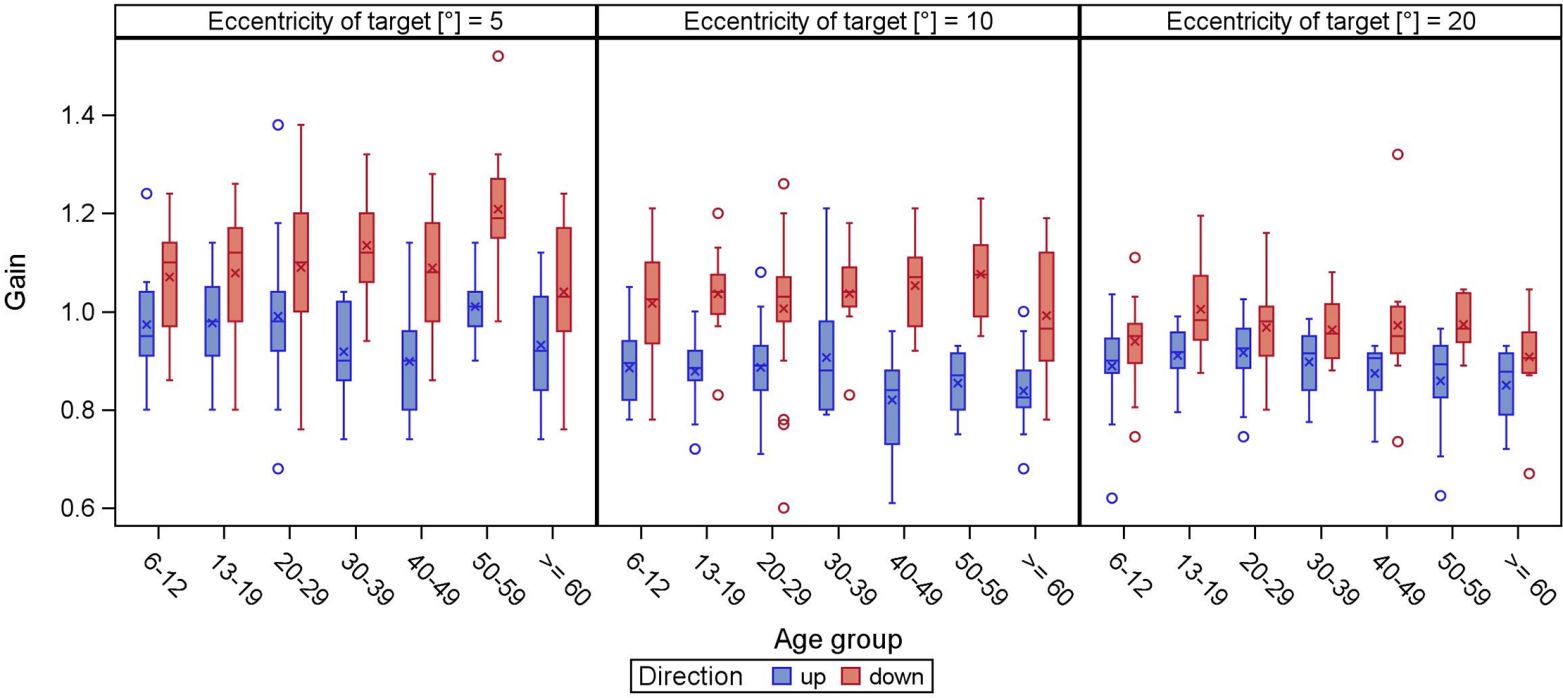
**Gain of vertical (direction up/down) reflexive saccades.** The gain (y-axis) is displayed in boxplots in dependence of the age groups (x-axis) for different target eccentricities.

Overall, 5°-saccades were mainly isometric, larger target displacements usually resulted in hypometric saccades (Figs 3 and 4). While this finding is held true for horizontal and upward saccades, downward saccades were either hyper- or isometric. In general, the larger the target displacement, the more gain decreased.

**Fig 5:**
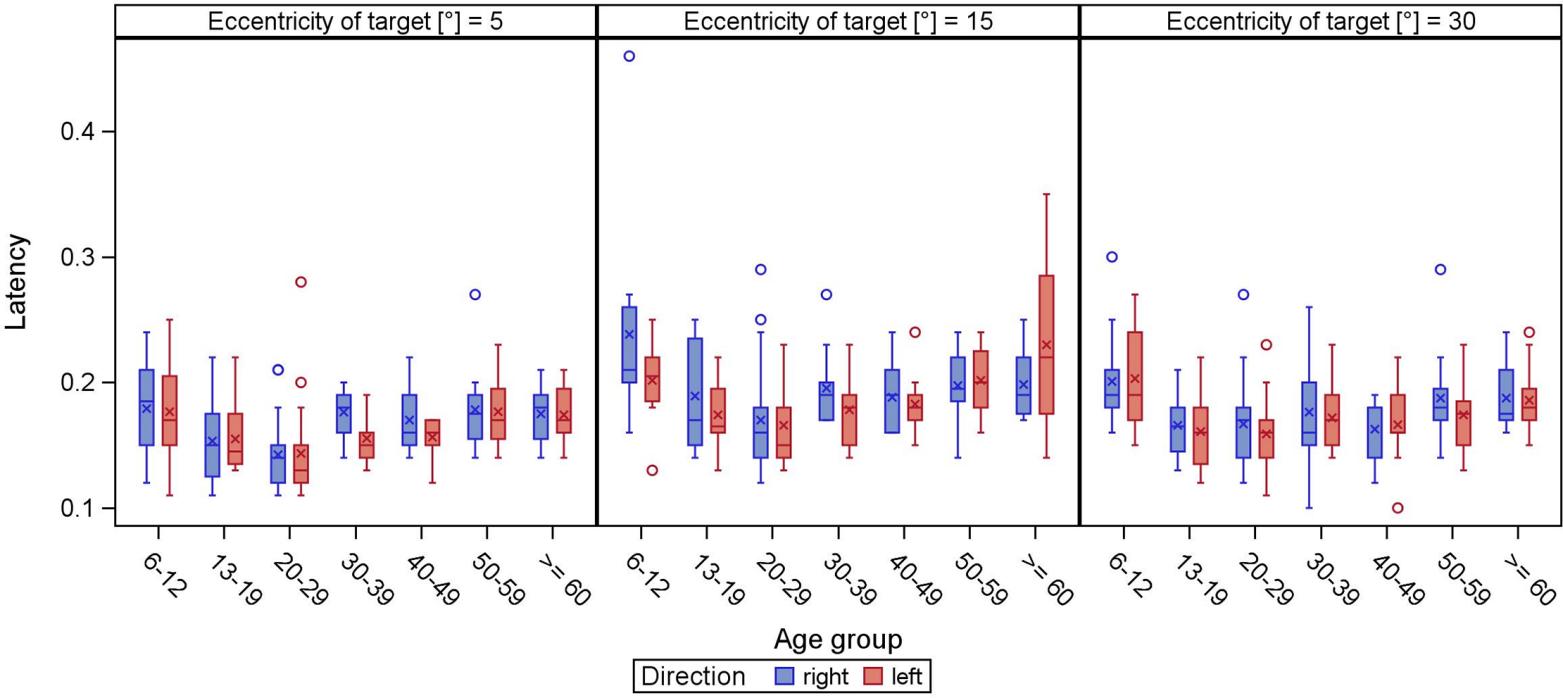
**Latency of horizontal (direction right/left) reflexive saccades.** The latency (y-axis) is displayed in boxplots in dependence of the age groups (x-axis) for different target eccentricities.

**Fig 6:**
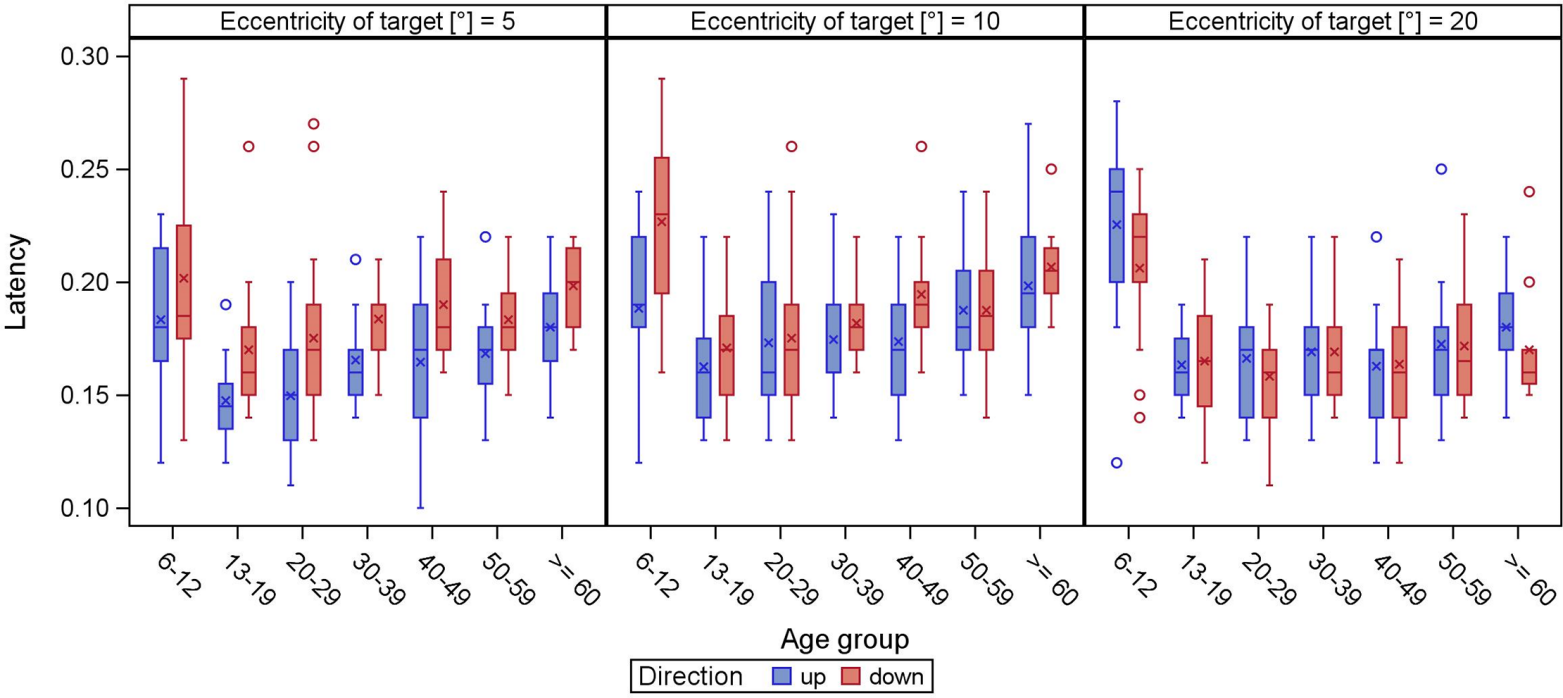
**Latency of vertical (direction up/down) reflexive saccades.** The latency (y-axis) is displayed in boxplots in dependence of the age groups (x-axis) for different target eccentricities.

Latency becomes shorter during childhood and adolescence, while in adulthood it is relatively stable with a slight trend to increase in the elderly (Figs 5 and 6).

### Influence of age, direction and eccentricity on saccadic peak velocity, gain and latency

When fitting a linear mixed model to the horizontal peak velocity we found significant effects of direction (p = 0.0225) with targets to the left showing on average 9.6°/s higher velocity than targets to the right. Eccentricity also had a significant (p<0.0001) effect on horizontal velocity: when the fixation target was at 30°, the velocity was on average 242.6°/s higher than for fixation target at 5°. For fixation target at 15°, the average velocity was 139.0°/s higher than for fixation target at 5°. Age was not significantly associated with horizontal velocity (p = 0.8653), with an estimated yearly decrease of only 0.0197°/s (S2 Table). The results regarding vertical peak velocity were almost similar (S3 Table). Only the effect of age on vertical peak velocity was different with a significant p-value (p = 0.0012), however the estimated yearly decrease was minuscule (0.4°/s) (S3 Table).

When fitting the linear mixed model to the horizontal and vertical gain we found significant effects of eccentricity (p<0.0001): with 0.13 lower gain for fixation targets at 15° or 30° horizontally than for fixation targets at 5°, and vertically with 0.08 and 0.11 lower gain for fixation targets at 10° or 20° respectively compared to fixation targets at 5° (S4 and S5 Tables). Age was not significantly associated with vertical gain (p = 0.0678), but with horizontal gain (p = 0.0191) however with an ignorable estimated yearly change (S4 and S5 Tables). Furthermore, age was not significantly associated with vertical latency (p = 0.7124) and the significant association of age with horizontal latency (p = 0.0248) with an estimated yearly increase of 0.183 ms per year of age was marginal (S6 and S7 Tables).

## Discussion

The main saccade parameters peak velocity and gain were found to be independent from age. In contrast, saccadic latency is shortest in young adults. The present data can serve as comparative normative cohort for studies using a similar device and examination protocol. Stratification or individual matching for further studies when focusing on peak velocity and gain is not required under the condition of identical direction of gaze and angular distance of target displacement. These findings are in contrast to formerly published data showing a negative correlation between age and peak velocity using a mobile EyeSeeCam device in a group of 34 individuals between 25 and 85 years of age [23]. For the first time, well standardized, normative data of reflexive saccades recorded by the EyeSeeCam are presented in this study, particularly since previous data bases were obtained by other devices. Furthermore, only limited data is available on saccades in children and young adults can be found, which make these normative data especially valuable.

The vast majority of saccades tested were iso- or slightly hypometric, with the exception of downgaze, which is characterized by hyper- or isometric saccades. This is in line with previously published data [6]. In contrast to our results, Bucci and Collewijn postulated less accurate downward saccades [24,25].

While saccadic latency was fairly stable in an age range from 13 to 88 years, we saw longer latencies in the subgroup from 6 to 12 years (around 0.21 s; 15° horizontally). These results are in line with a study analyzing saccades in children, mentioning not only decreasing latency in patients aged 8 to 19 years but also a mean of 258 ms (15° to the nose) in visually guided saccades [26]. Increased saccadic latency with advancing age is a common finding in the literature [11,12,15], although the reason is not clear. As mentioned by Irving et al., this might be explained by the development and ageing of the brain structures which control latency [14]. Our study shows a broad range of latencies. As latency involves visual processing, target choice and motor programming [6], we do not assume that age alone accounts for this variability. Other authors stress the fact that it is a cognitive-physiological parameter [24]. The latency means in this study (ranged from 160 to 190 ms) are all slightly faster as the typical latency of around 200 ms; they can be classified as fast regular saccades (between 120 and 200 ms), whereas express saccades have an even shorter latency of 80 to 120 ms [27]. It has to be emphasized that the latencies measured in the present study were only assessed by a standard protocol. We cannot exclude impact on this specific parameter. Additional protocols would be needed to rule out a possible interference of measurement strategy on latency.

The strength of this study is the broad age range and the standardized measurement set-up, which was kept stable for these 100 study participants measured by two investigators in all. Moreover, we used a protocol investigating different target displacements and gaze directions, while many other studies only report findings of one target category.

A limitation of our study is the fact that saccadometric measurements were carried out without refractive correction for near fixation. Though near/presbyopic correction is not to be a source of reduced data quality or longer latency, increased latency has been reported for saccades in amblyopic eyes [28]. Thus we cannot exclude that presbyopia might likewise interfere with latency as well as with precision.

Our participant under anticonvulsant medication was the eldest in this study and yet exhibited the fastest peak velocity over all. A thorough investigation of saccadometry in patients under neurotropic drugs would be highly desirable, especially considering the fact that patients suffering from neurodegenerative diseases or oculomotor apraxia may be on medication, too.

In summary, infrared-saccadometry at a 220 Hz sampling provides reliable results. The calibration prior to the examination is feasible for a trained investigator, although children are more difficult to handle during calibration and the following measurements, as their span of attention is limited and keeping the head in a fix position is more challenging. Nevertheless, feasibility of saccadometry in a clinical setting as described in the present study was shown in a representative cohort and over a broad age range.

These data may serve as normative control for future studies on expected abnormal saccades, especially for neurodegenerative disorders whether inherited (such as Gaucher disease type 3, Niemann-Pick disease type C and Huntington’s disease) or acquired (Parkinson’s disease, frontotemporal dementia and stroke). Within these diseases, reflexive saccades are characterized by slowed peak velocity, less precision and longer latencies in a typical pattern depending on the direction of the eye movements. Saccadometry has been reported to enable non-invasive screening/monitoring of the natural course and of the effects of novel therapeutic approaches likewise. The means of peak velocity and gain can be used independently from age respecting the angular distance of target displacement. Latency is susceptible to age, but very likely also dependent from factors such as attention and experimental setting. We suggest to control for IQ and further cognitive tests as well as for circulatory parameters in the future.

## Supporting information

S1 Table Main saccade parameters per direction and target displacement. (DOCX)

S2 Table Linear mixed model with horizontal peak velocity as dependent variables, age as quantitative fixed effect, eccentricity and direction as categorical fixed effects and subject as random effect. (DOCX)

S3 Table Linear mixed model with vertical peak velocity as dependent variable, age as quantitative fixed effect, eccentricity and direction as categorical fixed effects and subject as random effect. (DOCX)

S4 Table Linear mixed model with horizontal gain as dependent variable, age as quantitative fixed effect, eccentricity and direction as categorical fixed effects and subject as random effect. (DOCX)

S5 Table Linear mixed model with vertical gain as dependent variable, age as quantitative fixed effect, eccentricity and direction as categorical fixed effects and subject as random effect. (DOCX)

S6 Table Linear mixed model with horizontal latency as dependent variable, age as quantitative fixed effect, eccentricity and direction as categorical fixed effects and subject as random effect. (DOCX)

S7 Table Linear mixed model for vertical latency as dependent variable, age as quantitative fixed effect, eccentricity and direction as categorical fixed effects and subject as random effect. (DOCX)

S1 Fig The test sequence settings of saccadometry. (DOCX)
