# Supplementary material for: Age dependent normative data of vertical and horizontal reflexive saccades
Source: PLoS One. 2018 Sep 18;13(9):e0204008. doi: 10.1371/journal.pone.0204008 (PMC6143243; doi:10.1371/journal.pone.0204008)
Supplement: S1 Table — (DOCX) [file pone.0204008.s001.docx]

**S1 Table. Main saccade parameters per direction and target displacement.**

| *Stimulus* | *Peak Velocity [°/s]* | *Gain* | *Latency [ms]* |
| --- | --- | --- | --- |
| R 5° | 206 (± 29), [146-268] | 0.99 (± 0.09), [0.78-1.32] | 0.16 (± 0.03), [0.11-.027] |
| R 15° | 339 (± 48), [231-472] | 0.84 (± 0.06), [0.66-0.97] | 0.19 (± 0.05), [0.12-0.46] |
| R 30° | 459 (± 67), [305-677] | 0.87 (± 0.06), [0.73-0.98] | 0.18 (± 0.03), [0.10-0.30] |
| L 5° | 219 (± 29), [150-299] | 1.02 (± 0.09), [0.76-1.26] | 0.16 (± 0.03), [0.11-0.28] |
| L 15° | 364 (± 52), [218-503] | 0.90 (± 0.07), [0.65-1.11] | 0.19 (± 0.04), [0.13-0.35] |
| L 30° | 451 (± 66), [259-645] | 0.87 (± 0.06), [0.68-1.02] | 0.17 (± 0.03), [0.10-0.27] |
|  |  |  |  |
| U 5° | 199 (± 33), [119-290] | 0.96 (± 0.12), [0.68-1.38] | 0.16 (± 0.03), [0.10-0.23] |
| U 10° | 285 (± 48), [192-421] | 0.87 (± 0.09), [0.61-1.21] | 0.18 (± 0.03), [0.12-0.27] |
| U 20° | 404 (± 74), [224-609] | 0.89 (± 0.08), [0.62-1.04] | 0.18 (± 0.03), [0.12-0.28] |
| D 5° | 217 (± 39), [134-327] | 1.10 (± 0.14), [0.76-1.52] | 0.18 (± 0.03), [0.13-0.29] |
| D 10° | 320 (± 51), [194-440] | 1.03 (± 0.11), [0.60-1.26] | 0.19 (± 0.03), [0.13-0.29] |
| D 20° | 378 (± 67), [216-554] | 0.96 (± 0.09), [0.67-1.32] | 0.17 (± 0.03), [0.11-0.25] |
