# Supplementary material for: Age dependent normative data of vertical and horizontal reflexive saccades
Source: PLoS One. 2018 Sep 18;13(9):e0204008. doi: 10.1371/journal.pone.0204008 (PMC6143243; doi:10.1371/journal.pone.0204008)
Supplement: S2 Table — (DOCX) [file pone.0204008.s002.docx]

**S2 Table. Linear mixed model with horizontal peak velocity as dependent variables, age as quantitative fixed effect, eccentricity and direction as categorical fixed effects and subject as random effect.**

| **Effect** | | | | **Regression coefficient (β)** | | | **SE(β)** | **DF** | **t Value** | **p-value** | **Limits of 95% confidence interval for regression coefficient** | |
| --- | --- | --- | --- | --- | --- | --- | --- | --- | --- | --- | --- | --- |
| **Intercept** | | | | 202.24 | | | 6.6582 | 590 | 30.37 | <.0001 | 189.16 | 215.31 |
| **AGE (per year)** | | | | -0.01966 | | | 0.1159 | 590 | -0.17 | 0.8653 | -0.2472 | 0.2079 |
| **Direction** | | | |  | | |  |  |  |  |  |  |
| Right (Reference) | | | | 0 | | | . | . | . | . | . | . |
| Left | | | | 9.6007 | | | 4.1965 | 590 | 2.29 | 0.0225 | 1.3588 | 17.8426 |
| **Eccentricity of target [°]** | | | |  | | |  |  |  |  |  |  |
| 5 (Reference) | | | | 0 | | | . | . | . | . | . | . |
| 15 | | | | 139.01 | | | 5.1483 | 590 | 27.00 | <.0001 | 128.90 | 149.12 |
| 30 | | | | 242.60 | | | 5.1355 | 590 | 47.24 | <.0001 | 232.51 | 252.68 |
| **Type 3 Tests of Fixed Effects** | | | | | |  |  |  |  |  |  |  |
| **Effect** | **Num DF** | **Den DF** | **F Value** | | **Pr > F** |  |  |  |  |  |  |  |
| **AGE** | 1 | 590 | 0.03 | | 0.8653 |  |  |  |  |  |  |  |
| **Direction** | 1 | 590 | 5.23 | | 0.0225 |  |  |  |  |  |  |  |
| **Eccentricity** | 2 | 590 | 1123.14 | | <.0001 |  |  |  |  |  |  |  |

**S2 Table. Linear mixed model with horizontal peak velocity as dependent variables, age as quantitative fixed effect, eccentricity and direction as categorical fixed effects and subject as random effect.** Regression coefficients with standard errors (SE), degrees of freedom (DF), p-values and 95% confidence intervals.
