# Supplementary material for: Age dependent normative data of vertical and horizontal reflexive saccades
Source: PLoS One. 2018 Sep 18;13(9):e0204008. doi: 10.1371/journal.pone.0204008 (PMC6143243; doi:10.1371/journal.pone.0204008)
Supplement: S3 Table — (DOCX) [file pone.0204008.s003.docx]

**S3 Table. Linear mixed model with vertical peak velocity as dependent variable, age as quantitative fixed effect, eccentricity and direction as categorical fixed effects and subject as random effect.**

| **Effect** | | | | **Regression coefficient (β)** | | | **SE(β)** | **DF** | **t Value** | **p-value** | **Limits of 95% confidence interval for regression coefficient** | |
| --- | --- | --- | --- | --- | --- | --- | --- | --- | --- | --- | --- | --- |
| **Intercept** | | | | 207.71 | | | 7.2554 | 590 | 28.63 | <.0001 | 193.46 | 221.96 |
| **AGE (per year)** | | | | -0.4032 | | | 0.1243 | 590 | -3.24 | 0.0012 | -0.6474 | -0.1590 |
| **Direction** | | | |  | | |  |  |  |  |  |  |
| Up (Reference) | | | | 0 | | | . | . | . | . | . | . |
| Down | | | | 9.0805 | | | 4.5014 | 590 | 2.02 | 0.0441 | 0.2398 | 17.9213 |
| **Eccentricity of target [°]** | | | |  | | |  |  |  |  |  |  |
| 5 (Reference) | | | | 0 | | | . | . | . | . | . | . |
| 10 | | | | 94.6869 | | | 5.5223 | 590 | 17.15 | <.0001 | 83.8410 | 105.53 |
| 20 | | | | 183.05 | | | 5.5087 | 590 | 33.23 | <.0001 | 172.23 | 193.86 |
| **Type 3 Tests of Fixed Effects** | | | | | |  |  |  |  |  |  |  |
| **Effect** | **Num DF** | **Den DF** | **F Value** | | **Pr > F** |  |  |  |  |  |  |  |
| **AGE** | 1 | 590 | 10.52 | | 0.0012 |  |  |  |  |  |  |  |
| **Direction** | 1 | 590 | 4.07 | | 0.0441 |  |  |  |  |  |  |  |
| **Eccentricity** | 2 | 590 | 552.24 | | <.0001 |  |  |  |  |  |  |  |

**S3 Table. Linear mixed model with vertical peak velocity as dependent variable, age as quantitative fixed effect, eccentricity and direction as categorical fixed effects and subject as random effect.** Regression coefficients with standard errors (SE), degrees of freedom (DF), p-values and 95% confidence intervals.
