# Supplementary material for: Age dependent normative data of vertical and horizontal reflexive saccades
Source: PLoS One. 2018 Sep 18;13(9):e0204008. doi: 10.1371/journal.pone.0204008 (PMC6143243; doi:10.1371/journal.pone.0204008)
Supplement: S4 Table — (DOCX) [file pone.0204008.s004.docx]

**S4 Table. Linear mixed model with horizontal gain as dependent variable, age as quantitative fixed effect, eccentricity and direction as categorical fixed effects and subject as random effect.**

| **Effect** | | | | **Regression coefficient (β)** | | | **SE(β)** | **DF** | **t Value** | **p-value** | **Limits of 95% confidence interval for regression coefficient** | |
| --- | --- | --- | --- | --- | --- | --- | --- | --- | --- | --- | --- | --- |
| **Intercept** | | | | 0.9759 | | | 0.008151 | 590 | 119.73 | <.0001 | 0.9599 | 0.9919 |
| **AGE (per year)** | | | | 0.000384 | | | 0.000163 | 590 | 2.35 | 0.0191 | 0.000063 | 0.000705 |
| **Direction** | | | |  | | |  |  |  |  |  |  |
| Right (Reference) | | | | 0 | | | . | . | . | . | . | . |
| Left | | | | 0.02964 | | | 0.005933 | 590 | 5.00 | <.0001 | 0.01799 | 0.04129 |
| **Eccentricity of target [°]** | | | |  | | |  |  |  |  |  |  |
| 5 (Reference) | | | | 0 | | | . | . | . | . | . | . |
| 15 | | | | -0.1312 | | | 0.007279 | 590 | -18.03 | <.0001 | -0.1455 | -0.1170 |
| 30 | | | | -0.1306 | | | 0.007261 | 590 | -17.98 | <.0001 | -0.1448 | -0.1163 |
| **Type 3 Tests of Fixed Effects** | | | | | |  |  |  |  |  |  |  |
| **Effect** | **Num DF** | **Den DF** | **F Value** | | **Pr > F** |  |  |  |  |  |  |  |
| **AGE** | 1 | 590 | 5.52 | | 0.0191 |  |  |  |  |  |  |  |
| **Direction** | 1 | 590 | 24.96 | | <.0001 |  |  |  |  |  |  |  |
| **Eccentricity** | 2 | 590 | 215.98 | | <.0001 |  |  |  |  |  |  |  |

**S4 Table. Linear mixed model with horizontal gain as dependent variable, age as quantitative fixed effect, eccentricity and direction as categorical fixed effects and subject as random effect.** Regression coefficients with standard errors (SE), degrees of freedom (DF), p-values and 95% confidence intervals.
