# Supplementary material for: Age dependent normative data of vertical and horizontal reflexive saccades
Source: PLoS One. 2018 Sep 18;13(9):e0204008. doi: 10.1371/journal.pone.0204008 (PMC6143243; doi:10.1371/journal.pone.0204008)
Supplement: S5 Table — (DOCX) [file pone.0204008.s005.docx]

**S5 Table. Linear mixed model with vertical gain as dependent variable, age as quantitative fixed effect, eccentricity and direction as categorical fixed effects and subject as random effect.**

| **Effect** | | | | **Regression coefficient (β)** | | | **SE(β)** | **DF** | **t Value** | **p-value** | **Limits of 95% confidence interval for regression coefficient** | |
| --- | --- | --- | --- | --- | --- | --- | --- | --- | --- | --- | --- | --- |
| **Intercept** | | | | 0.9862 | | | 0.01210 | 590 | 81.49 | <.0001 | 0.9624 | 1.0100 |
| **AGE (per year)** | | | | -0.00044 | | | 0.000243 | 590 | -1.83 | 0.0678 | -0.00092 | 0.000033 |
| **Direction** | | | |  | | |  |  |  |  |  |  |
| Up (Reference) | | | | 0 | | | . | . | . | . | . | . |
| Down | | | | 0.1208 | | | 0.008809 | 590 | 13.71 | <.0001 | 0.1035 | 0.1381 |
| **Eccentricity of target [°]** | | | |  | | |  |  |  |  |  |  |
| 5 (Reference) | | | | 0 | | | . | . | . | . | . | . |
| 10 | | | | -0.08318 | | | 0.01081 | 590 | -7.70 | <.0001 | -0.1044 | -0.06196 |
| 20 | | | | -0.1054 | | | 0.01078 | 590 | -9.78 | <.0001 | -0.1266 | -0.08426 |
| **Type 3 Tests of Fixed Effects** | | | | | |  |  |  |  |  |  |  |
| **Effect** | **Num DF** | **Den DF** | **F Value** | | **Pr > F** |  |  |  |  |  |  |  |
| **AGE** | 1 | 590 | 3.35 | | 0.0678 |  |  |  |  |  |  |  |
| **Direction** | 1 | 590 | 187.95 | | <.0001 |  |  |  |  |  |  |  |
| **Eccentricity** | 2 | 590 | 53.04 | | <.0001 |  |  |  |  |  |  |  |

**S5 Table. Linear mixed model with vertical gain as dependent variable, age as quantitative fixed effect, eccentricity and direction as categorical fixed effects and subject as random effect.** Regression coefficients with standard errors (SE), degrees of freedom (DF), p-values and 95% confidence intervals.
