# Supplementary material for: Age dependent normative data of vertical and horizontal reflexive saccades
Source: PLoS One. 2018 Sep 18;13(9):e0204008. doi: 10.1371/journal.pone.0204008 (PMC6143243; doi:10.1371/journal.pone.0204008)
Supplement: S6 Table — (DOCX) [file pone.0204008.s006.docx]

**S6 Table. Linear mixed model with horizontal latency as dependent variable, age as quantitative fixed effect, eccentricity and direction as categorical fixed effects and subject as random effect.**

| **Effect** | | | | **Regression coefficient (β)** | | | **SE(β)** | **DF** | **t Value** | **p-value** | **Limits of 95% confidence interval for regression coefficient** | |
| --- | --- | --- | --- | --- | --- | --- | --- | --- | --- | --- | --- | --- |
| **Intercept** | | | | 0.1465 | | | 0.004826 | 590 | 30.37 | <.0001 | 0.1371 | 0.1560 |
| **AGE (per year)** | | | | 0.000183 | | | 0.000081 | 590 | 2.25 | 0.0248 | 0.000023 | 0.000343 |
| **Direction** | | | |  | | |  |  |  |  |  |  |
| Right (Reference) | | | | 0 | | | . | . | . | . | . | . |
| Left | | | | -0.00470 | | | 0.002947 | 590 | -1.59 | 0.1114 | -0.01049 | 0.001089 |
| **Eccentricity of target [°]** | | | |  | | |  |  |  |  |  |  |
| 5 (Reference) | | | | 0 | | | . | . | . | . | . | . |
| 15 | | | | 0.02803 | | | 0.003615 | 590 | 7.75 | <.0001 | 0.02093 | 0.03513 |
| 30 | | | | 0.01309 | | | 0.003606 | 590 | 3.63 | 0.0003 | 0.006006 | 0.02017 |
| **Type 3 Tests of Fixed Effects** | | | | | |  |  |  |  |  |  |  |
| **Effect** | **Num DF** | **Den DF** | **F Value** | | **Pr > F** |  |  |  |  |  |  |  |
| **AGE** | 1 | 590 | 5.06 | | 0.0248 |  |  |  |  |  |  |  |
| **Direction** | 1 | 590 | 2.54 | | 0.1114 |  |  |  |  |  |  |  |
| **Eccentricity** | 2 | 590 | 30.10 | | <.0001 |  |  |  |  |  |  |  |

**S6 Table. Linear mixed model with horizontal latency as dependent variable, age as quantitative fixed effect, eccentricity and direction as categorical fixed effects and subject as random effect.** Regression coefficients with standard errors (SE), degrees of freedom (DF), p-values and 95% confidence intervals.
