# Supplementary material for: Age dependent normative data of vertical and horizontal reflexive saccades
Source: PLoS One. 2018 Sep 18;13(9):e0204008. doi: 10.1371/journal.pone.0204008 (PMC6143243; doi:10.1371/journal.pone.0204008)
Supplement: S7 Table — (DOCX) [file pone.0204008.s007.docx]

**S7 Table. Linear mixed model for vertical latency as dependent variable, age as quantitative fixed effect, eccentricity and direction as categorical fixed effects and subject as random effect.**

| **Effect** | | | | **Regression coefficient (β)** | | | **SE(β)** | **DF** | **t Value** | **p-value** | **Limits of 95% confidence interval for regression coefficient** | |
| --- | --- | --- | --- | --- | --- | --- | --- | --- | --- | --- | --- | --- |
| **Intercept** | | | | 0.1590 | | | 0.004174 | 590 | 38.08 | <.0001 | 0.1508 | 0.1672 |
| **AGE (per year)** | | | | 0.000026 | | | 0.000070 | 590 | 0.37 | 0.7124 | -0.00011 | 0.000164 |
| **Direction** | | | |  | | |  |  |  |  |  |  |
| Up (Reference) | | | | 0 | | | . | . | . | . | . | . |
| Down | | | | 0.008591 | | | 0.002549 | 590 | 3.37 | 0.0008 | 0.003584 | 0.01360 |
| **Eccentricity of target [°]** | | | |  | | |  |  |  |  |  |  |
| 5 (Reference) | | | | 0 | | | . | . | . | . | . | . |
| 10 | | | | 0.01040 | | | 0.003127 | 590 | 3.33 | 0.0009 | 0.004262 | 0.01655 |
| 20 | | | | -0.00052 | | | 0.003120 | 590 | -0.17 | 0.8680 | -0.00665 | 0.005608 |
| **Type 3 Tests of Fixed Effects** | | | | | |  |  |  |  |  |  |  |
| **Effect** | **Num DF** | **Den DF** | **F Value** | | **Pr > F** |  |  |  |  |  |  |  |
| **AGE** | 1 | 590 | 0.14 | | 0.7124 |  |  |  |  |  |  |  |
| **Direction** | 1 | 590 | 11.36 | | 0.0008 |  |  |  |  |  |  |  |
| **Eccentricity** | 2 | 590 | 7.78 | | 0.0005 |  |  |  |  |  |  |  |

**S7 Table. Linear mixed model for vertical latency as dependent variable, age as quantitative fixed effect, eccentricity and direction as categorical fixed effects and subject as random effect.** Regression coefficients with standard errors (SE), degrees of freedom (DF), p-values and 95% confidence intervals.
