# Supplementary material for: Age dependent normative data of vertical and horizontal reflexive saccades
Source: PLoS One. 2018 Sep 18;13(9):e0204008. doi: 10.1371/journal.pone.0204008 (PMC6143243; doi:10.1371/journal.pone.0204008)
Supplement: S1 Fig — (DOCX) [file pone.0204008.s008.docx]

**S1 Fig. The test sequence settings of saccadometry.**


**S1 Fig. The test sequence settings of saccadometry.** Eye position (y-axis) in dependence of time (x-axis) during the sequence, the black line displaying the stimulus track and the blue line representing the eye track, green and red dots mark the beginning and ending of a saccade respectively (in this case the measurement was slightly abnormal as it was a Gaucher patient’s saccadometry).
